# Lupus nephritis: management challenges during pregnancy

**DOI:** 10.3389/fneph.2024.1390783

**Published:** 2024-06-04

**Authors:** Zohreh Gholizadeh Ghozloujeh, Tripti Singh, Kenar D. Jhaveri, Silvi Shah, Edgar Lerma, Amir Abdipour, Sayna Norouzi

**Affiliations:** ^1^ Department of Medicine, Division of Nephrology, Loma Linda University School of Medicine, Loma Linda, CA, United States; ^2^ Department of Medicine, Division of Nephrology, University of Wisconsin, Madison, WI, United States; ^3^ Department of Medicine, Division of Kidney Diseases and Hypertension, Zucker School of Medicine at Hofstra/Northwell, Northwell Health, Great Neck, NY, United States; ^4^ Department of Medicine, Division of Nephrology, University of Cincinnati College of Medicine, Cincinnati, OH, United States; ^5^ Department of Medicine, Division of Nephrology, Advocate Christ Medical Center, University of Illinois at Chicago, Oak Lawn, IL, United States

**Keywords:** lupus nephritis, pregnancy, management of pregnancy, pre-conception counseling, multidisciplinary management

## Abstract

Lupus nephritis (LN), a severe complication of systemic lupus erythematosus (SLE), leads to significant kidney inflammation and damage and drastically increases mortality risk. Predominantly impacting women in their reproductive years, LN poses specific risks during pregnancy, including pre-eclampsia, growth restrictions, stillbirth, and preterm delivery, exacerbated by lupus activity, specific antibodies, and pre-existing conditions like hypertension. Effective management of LN during pregnancy is crucial and involves carefully balancing disease control with the safety of the fetus. This includes pre-conception counseling and a multidisciplinary approach among specialists to navigate the complexities LN patients face during pregnancy, such as distinguishing LN flare-ups from pregnancy-induced conditions. This review focuses on exploring the complex dynamics between pregnancy and LN, emphasizing the management difficulties and the heightened risks pregnant women with LN encounter.

## Introduction

Lupus nephritis (LN) is a severe manifestation of systemic lupus erythematosus (SLE), characterized by inflammation and kidney damage. Prevalence rates in SLE patients range from 20% to 65%, with LN associated with higher mortality compared to the general population (1–3). It predominantly affects women in their reproductive years, presenting unique challenges during pregnancy (4). Pregnant women with LN are at increased risk for complications like pre-eclampsia, intrauterine growth restriction, stillbirth, and preterm delivery (5). The risk is further amplified by factors such as lupus activity, the presence of specific antibodies (like Antiphospholipid antibody (APA), anti-SSA/Ro, and anti-SSB/La), and pre-existing conditions like hypertension (6). The differentiation between an LN flare and pregnancy-induced hypertension or proteinuria can be challenging but is crucial for appropriate management.

Lupus nephritis involves the deposition of immune complexes in the kidneys, triggering a cascade of inflammatory responses, including local complement activation, leukocyte recruitment, and cytokine signaling, leading to glomerular and tubulointerstitial injury. This inflammatory process leads to kidney damage presenting as proteinuria, acute kidney injury, chronic kidney disease (CKD), and eventually, in some cases, end-stage kidney disease (ESKD).

The progression of LN to ESKD is influenced by various factors, including the initial nephron number (determined at birth), episodes of nephron loss (during LN flare), and the rate of nephron loss beyond physiological aging (ongoing inflammation due to LN) or in some cases concomitant uncontrolled diabetes mellitus, and/or hypertension (6). Flares of LN are episodes of acute kidney injury that result in irreversible nephron loss, shortening the kidney lifespan. Persistent or smoldering LN, even with induction therapy, accelerates the annual rate of nephron loss, hastening the onset of ESKD (7, 8).

Effective management of LN during pregnancy is pivotal to minimizing maternal and fetal risks. Tailoring treatment strategies that balance disease control and fetal safety is imperative. Pre-conception counseling and multidisciplinary care involving rheumatologists, nephrologists, and obstetricians are vital to optimize outcomes. Monitoring and managing blood pressure, kidney function, and lupus activity throughout pregnancy and postpartum safeguard maternal and fetal health (**Table 1**). The management strategy should also consider the potential impact of medications on the fetus and adjust them accordingly to ensure safety while maintaining disease control.

**Table 1:** Preconception to postpartum: managing lupus nephritis during maternal transition.

Stage	Recommendations
Preconception	- Conceive during a period of stable disease remission, preferably for at least 6 months. - Perform a thorough evaluation of disease activity and organ involvement. - Ensure SLE and LN are in remission or low activity; consider the SLEDAI score. - Test for anti-dsDNA antibodies and complement levels if there is an active disease or a history of flares. - Be aware of C4 levels as a risk factor for SLE flare and low C3 levels as a potential risk for preterm delivery.
During Pregnancy	- Advise on the likelihood of preterm delivery and increased risk of operative delivery. - Monitor LN activity closely with regular nephrologist assessments, more frequently in active SLE. - Conduct fetal echocardiography for anti-Ro/SSA and/or anti-La/SSB positive patients. - Differentiate between LN flare and preeclampsia; initiate therapy upon diagnosis of LN flare. - Be vigilant for preeclampsia development after 20 weeks of gestation in SLE patients. - Implement tailored maternal-fetal monitoring strategies, including frequent ultrasounds and fetal testing.
Postpartum	- Monitor for LN flare and thromboembolic events within the first six months post-delivery. - Encourage breastfeeding, discussing the safety of medications during lactation. - Follow-up closely postpartum, using prophylactic low-molecular-weight heparin for at-risk women. - Consider treatment adjustments for severe lupus or lupus nephritis, especially in cases of premature delivery.

This review aims to comprehensively explore the complex interplay between pregnancy and LN, shedding light on the increased risks and challenges encountered by pregnant women with LN.

## Preconception counseling and risk assessment for women with lupus nephritis

### Preconception counseling

Preconception counseling is pivotal for women with LN to navigate through the complexities of pregnancy safely. Biomarkers in Antiphospholipid Syndrome and Systemic Lupus Erythematosus (PROMISSE) study findings indicated that LN relapse rate of 7.8% of patients in complete remission and 21% of those in partial remission during pregnancy (9). Current evidence has shown that SLE patients without LN flare in the pre-conception period have a small risk of relapse during pregnancy (10–12). The reduction in SLE flare rates during pregnancy may be attributed to thorough pre-pregnancy counseling, diligent management, and continuous monitoring, which includes adjusting medications for maternal and fetal safety and identifying signs of disease activity early. This leads to the sustained use of suitable medications like hydroxychloroquine, effectively lowering flare risks (13). The optimal time for a woman with LN to conceive is generally during a period of stable disease remission, typically sustained for at least 6 months (14). Specifically, Attia et al. observed that only 12.5% of patients with inactive LN in the six months preceding conception experienced LN relapse during pregnancy. In contrast, this incidence was significantly higher (81.3%) in patients with active LN (15). However, the time between LN flare remission and the conception period could be shorter if the disease flare is mild, but a careful and individualized assessment is crucial.

## Assessing disease activity and organ involvement

Before conception, a thorough evaluation of disease activity and organ involvement is imperative. This involves:

- Disease Activity: Ensuring SLE and LN are in remission or low activity. The Systemic Lupus Erythematosus Disease Activity Index (SLEDAI) is a concise tool that assesses SLE activity over the previous ten days, incorporating clinical symptoms and laboratory findings such as skin rashes, arthritis, kidney function, neurological symptoms, blood abnormalities, and immunological markers (16) (**Table 2**). It offers a global score that reflects disease severity, proving to be an excellent, easy-to-use method with strong psychometric properties for evaluating disease activity (18). Higher scores indicate more active disease, guiding treatment decisions and monitoring progression or remission effectively. This score is essential in pregnancy, as it can help determine the risk level for pregnant patients with SLE and LN. A SLEDAI score of 4 or more or a history of LN six months before conception has been shown to predict adverse maternal outcomes (19).
- Kidney Function: Monitoring serum creatinine levels (Normal range: 0.6 to 1.1 mg/dL) urinalysis with urine sediment, and spot urine protein/creatinine ratio (A ratio of less than 0.2 mg/mg or <200 mg/g are considered normal).
- Other Organ Involvement: In addition to renal involvement, LN frequently affects other organ systems, notably the cardiovascular, neuropsychiatric, and pulmonary systems. To accurately assess and document the extent of tissue damage in these systems, specific diagnostic approaches are recommended:
- Pulmonary assessment: The utilization of chest X-ray, High resonance computed topography, pulmonary angiography, and pulmonary function tests is crucial in identifying conditions such as pulmonary hypertension, fibrosis, and pleural fibrosis.
- Cardiovascular assessment: Echocardiography serves as the primary tool for detecting abnormalities, including valvular diseases and pericarditis. In cases where there is suspicion or evidence of cardiovascular disease, further investigation through angiography may be warranted.
- Neuropsychiatric assessment: Paying attention to the patient’s current medications, particularly those targeting psychological or neurological symptoms. Active seizures and their treatment status are of particular concern, requiring thorough evaluation and management.
- Laboratory Testing: Including anti-Ro/SSA, anti-La/SSB antibodies, Complete blood count, liver function tests, anti-dsDNA antibodies, and complement levels.
- Antiphospholipid Antibodies: Evaluating for IgG and IgM anticardiolipin (aCL) antibodies, IgG and IgM anti-beta2 glycoprotein I (anti-beta2GPI) antibodies, and lupus anticoagulant (LA) especially if there is a history of thrombotic events or pregnancy-related complications.

**Table 2:** SLEDAI-2K score descriptors grouped by organ systems (17).

Organ System	Descriptor	Points
Neurological	Recent onset seizure	8
	Psychosis	8
	Organic brain syndrome	8
	New onset sensory or motor neuropathy involving cranial nerves	8
	Lupus headache	8
	New onset stroke	8
Dermatological	Inflammatory-type rash	2
	Alopecia	2
Musculoskeletal	Arthritis	4
	Myositis	4
Renal	Heme-granular or RBC urinary casts	4
	Hematuria (>5 RBC/high-power field)	4
	Proteinuria (>0.5 g/24 hours)	4
	Pyuria (>5 WBC/high-power field)	4
Cardiopulmonary	Pleuritic chest pain with pleural rub/effusion or pleural thickening	2
	Pericarditis	2
Vascular	Vasculitis	8
Ophthalmological	Visual disturbance	8
Immunological	Low complement (CH50, C3, C4 decreased below lower limit of normal for lab)	2
	High DNA binding (Increased above normal range for lab)	2
Hematological	Platelets <100 x 10^9/L	1
	WBC <3 x 10^9/L	1
Mucosal	Oral or nasal mucosal ulcers	2
General/Constitutional	Temp >100.4°F (38°C)	1

## Identifying potential risks and complications

Identifying and discussing potential risks and complications during pregnancy is crucial. This includes:

- LN Flares: Understanding that LN flares can occur during pregnancy and postpartum.
- Preeclampsia: Women with LN have an increased risk of developing preeclampsia and should be monitored closely.
- Fetal Risks: Discuss risks like preterm birth, intrauterine growth restriction, and neonatal lupus, especially if anti-Ro/SSA and/or anti-La/SSB antibodies are present.
- Medication Management: Ensuring medications are adjusted to be safe during pregnancy and breastfeeding while maintaining disease control.

## Medication management in preconception and during pregnancy

**Figure 1:**
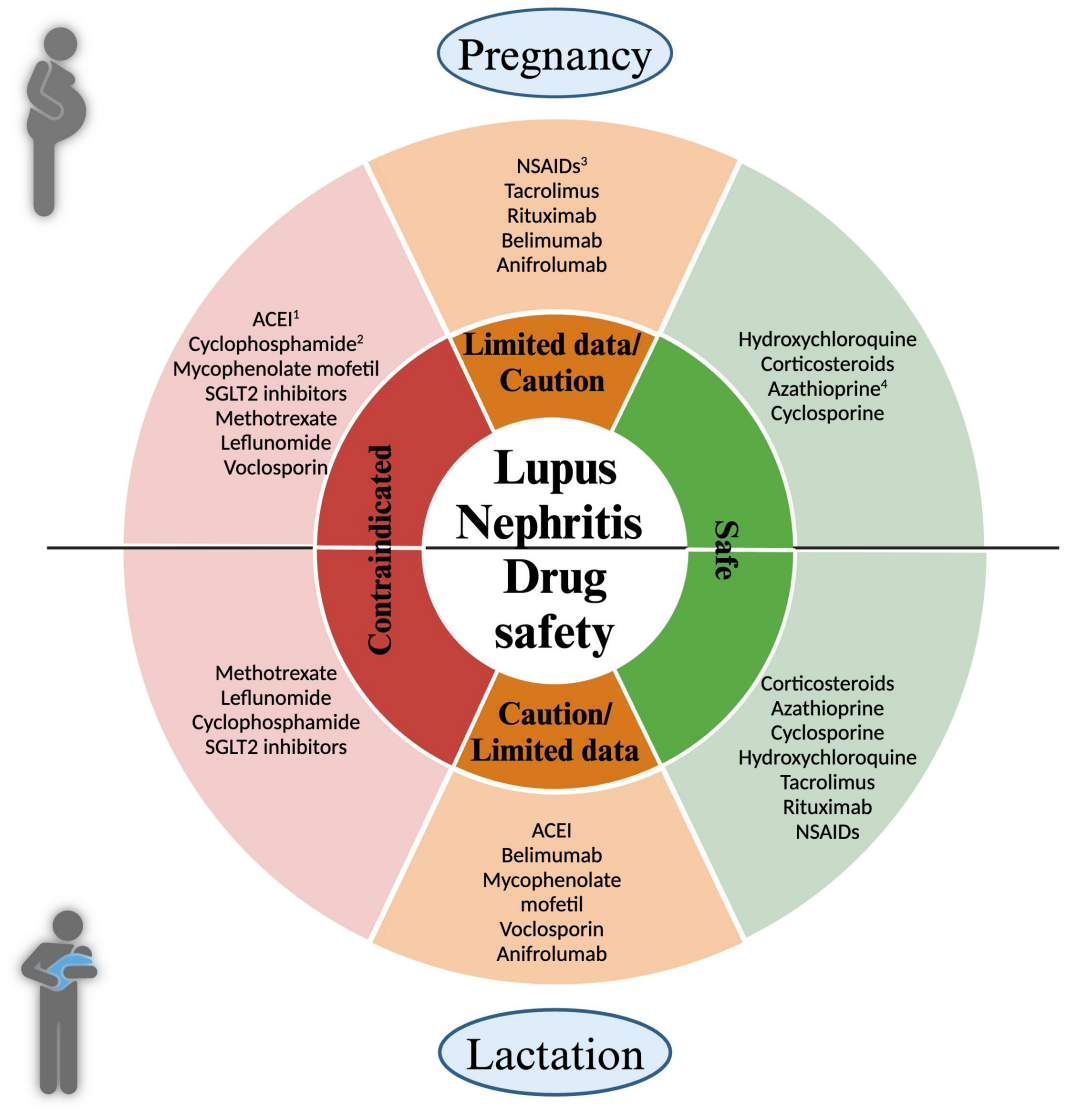
Medication Safety in Pregnancy and Lactation for Lupus Nephritis Patients. This figure delineates the safety of medications used in managing lupus nephritis during pregnancy (top half) and lactation (bottom half). Medications are categorized into three distinct groups: Green: safe; Orange: drugs requiring caution or with limited; Red: contraindicated. During pregnancy: 1. ACEI (Angiotensin-converting enzyme inhibitor): Contraindicated in the second and third trimester due to fetal renal effects; 2. Cyclophosphamide: Its use may be justified in severe relapses in the 2nd and 3rd trimester, 3. NSAIDs (Nonsteroidal Anti-inflammatory Drugs): Caution in the first trimester for women having difficulty conceiving; Increased risk of oligohydramnios after 20 weeks; 4. Azathioprine: Doses should not exceed 2 mg/kg/day.

Lupus nephritis management involves various medications to control disease activity and protect kidney function. Pregnancy necessitates a careful review of medications due to potential risks to the fetus. Medications should be adjusted to those that are safe during pregnancy while still effectively managing LN. Adjustments to medication regimens should be individualized, considering both the mother’s health and fetal safety. Active disease management during pregnancy might require modifying treatment plans while minimizing risks. A brief overview of the medications commonly utilized in the treatment of SLE and LN is provided in the following sections (**Figure 1**).

## Recommended during pregnancy

### Hydroxychloroquine

Hydroxychloroquine (HCQ) effectively reduces the risk of SLE during pregnancy, as demonstrated in multiple studies (20–23). Hydroxychloroquine also enhances renal remission rates and lowers the risk of renal flares in lupus nephritis patients. Studies have shown that patients with Class V lupus nephritis on HCQ have significantly higher rates of complete renal remission than those without HCQ (24).

This was supported by further evidence indicating that antimalarial treatment more than doubled the complete remission rates in Class V nephritis patients (25). Additionally, HCQ was associated with a 60% higher chance of achieving complete response in patients with proliferative forms of the disease (26). A retrospective cohort study also found an increased likelihood of renal recovery among lupus nephritis patients with renal impairment on HCQ (27). Moreover, a study on patients with quiescent lupus demonstrated that continuing HCQ reduced the risk of renal flares over three years (28).

However, while traditionally considered safe during pregnancy, a study revealed a slight elevation in the risk of congenital anomalies associated with a daily HCQ dosage exceeding 400 mg in the first trimester. However, this correlation did not emerge when the dosage remained below 400 mg (29). Additionally, there’s potential evidence for reducing the incidence of congenital heart block among at-risk fetuses from mothers with anti-Ro/SSA and anti-La/SSB antibodies when exposed to HCQ (30). In addition to the previously discussed studies, recent research has further reinforced the favorable impact of hydroxychloroquine (HCQ) on pregnancy outcomes in women with lupus nephritis. The study by Balevic et al. demonstrated that maintaining HCQ adherence significantly reduced the odds of preterm birth, highlighting the importance of medication compliance (31). Additionally, findings from Rahman et al. underscored the broader benefits of HCQ, revealing improved neonatal outcomes and a reduction in adverse pregnancy outcomes and antenatal complications such as hypertension and diabetes (32). Together, these studies provide compelling evidence for the continued use of HCQ in pregnant individuals with lupus nephritis to optimize both maternal and neonatal health.

### Low-dose aspirin

In the management of SLE during pregnancy, the initiation of low-dose aspirin around the 12th week of gestation is recommended to minimize the risk of preeclampsia and its complications, irrespective of the presence of antiphospholipid antibodies (aPLs). This approach, endorsed by the United States Preventive Services Task Force, is particularly crucial for pregnant women at high risk for preeclampsia, a category that includes those with SLE (33).

Recent studies further contribute to understanding aspirin’s role during pregnancy in SLE patients. A comprehensive analysis by Tani et al., involving 216 pregnancies, found that while initiating low-dose aspirin did not significantly alter overall complication rates, it showed promise in potentially lowering the frequency of pre-eclampsia (34). Additionally, Zhang et al.’s study demonstrated that combining hydroxychloroquine with aspirin significantly improved pregnancy outcomes, suggesting aspirin as a valuable adjunctive therapy for pregnant women with SLE (34, 35).

## Selective use allowed during pregnancy

### Nonsteroidal anti-inflammatory drugs

NSAIDs pose considerations during pregnancy, with evidence showing no link to congenital anomalies (36–38). However, uncertainty persists regarding their association with spontaneous abortion in the first trimester (39, 40). Caution is advised beyond 20 weeks due to a small risk of oligohydramnios (41).

The FDA recommends the lowest NSAID dose between 20 and 30 weeks, with avoidance after 30 weeks to prevent complications, including premature closure of the ductus arteriosus (42, 43).

Generally, NSAIDs, including Cox-1 and Cox-2 inhibitors, are discouraged in the third trimester due to heightened risks of adverse outcomes like ductus arteriosus closure.

In the postpartum period, the use of NSAIDs does not lead to an increase in blood pressure in women who have experienced hypertensive disorders during pregnancy (44, 45).

### Glucocorticoids

Glucocorticoids may be cautiously used during pregnancy to manage lupus nephritis flares if clinically appropriate (46, 47). It is widely acknowledged that treating severe diseases may warrant higher doses. Inflammation resulting from unmanaged autoimmune conditions can be more detrimental to the health of both mother and fetus than the impact of high-dose steroids (45). A study by Shimada et al. showed that an even lower dose of glucocorticoid (mean dose of 6.5 mg/day) during pregnancy was strongly linked to adverse pregnancy outcomes (48). This emphasizes the importance of carefully managing glucocorticoid use in SLE patients during pregnancy to mitigate these risks.

### Azathioprine

Azathioprine (AZA) is considered a suitable option during pregnancy when benefits outweigh potential risks. Comprehensive studies have not shown a significant correlation between AZA use during pregnancy and the occurrence of congenital anomalies (49, 50). It is essential to note that AZA is compatible with pregnancy (5), but caution should be exercised to ensure that doses do not exceed 2 mg/kg/day. Despite these considerations, AZA is generally recommended to be continued during pregnancy.

### Cyclosporine

Preliminary data indicate that children who were exposed to cyclosporine *in utero* have normal kidney function. However, its administration during pregnancy should be limited to situations where the maternal benefit outweighs potential risks to the fetus (51, 52). Classified as FDA category C, cyclosporine may be considered an acceptable alternative in specific circumstances where the potential benefits justify the potential risks. Its use should be carefully evaluated, particularly in autoimmune diseases refractory to conventional treatment (53).

### Tacrolimus

Tacrolimus, employed in both induction and maintenance therapy for lupus nephritis, has demonstrated effectiveness during pregnancy (54). A recently published case series reported successful management of lupus nephritis flares with tacrolimus in pregnant individuals. In another study involving 54 deliveries in SLE patients, comparable pregnancy outcomes were observed between those exposed to tacrolimus and those who were not (55).

Despite the limited number of fetuses exposed, a definitive causal link between tacrolimus use and congenital anomalies has not been established. Reports on using tacrolimus to treat lupus nephritis during pregnancy are limited, but a small study indicated that mothers on tacrolimus had healthy newborns (56).

### Antihypertensive medications

Methyldopa, nifedipine, and labetalol are safe antihypertensive options in pregnant patients (57, 58). In contrast, the use of angiotensin-converting enzyme (ACE) inhibitors and angiotensin II receptor blockers is unequivocally contraindicated during pregnancy due to the increased risk of adverse pregnancy outcomes associated with their administration (59). While diuretics may be considered with caution, careful and deliberate use is imperative.

## Selective use with caution in pregnancy

### Biologic medications

The use of biologic medications, like rituximab, during pregnancy is constrained by limited data. Given that IgG does not significantly cross the placenta until the 15th week of gestation, these medications can be continued through conception. In cases of maintenance treatment, rituximab is often strategically administered just before conception or in the first trimester (60, 61). Notably, for severe lupus nephritis flares, rituximab is increasingly considered, with the RITUXILUP study suggesting a potential role as a steroid-sparing agent (62). Additionally, it should not be used in the third trimester due to the risk of neonatal B cell depletion (63). However, caution is advised, and further research is warranted to assess risks and benefits comprehensively.

Belimumab, a novel BAFF inhibitor recently introduced, has undergone studies during pregnancy. Current research has not indicated an increased risk of fetal anomalies or severe infection (64–66). Nevertheless, cautious monitoring is advised, and additional data are required to validate its safety in pregnant patients fully.

## Contraindicated in pregnancy

### Cyclophosphamide

is associated with fetal loss and congenital malformations. Therefore, it is recommended to refrain from its use during the first 10 weeks of gestation, a period marked by heightened fetal susceptibility to teratogens (67). However, in life-threatening clinical scenarios, this medication has been employed during later stages of pregnancy (68).

### Mycophenolate mofetil

Avoidance of this medication is imperative during pregnancy due to its association with increased risks of first-trimester pregnancy loss and a heightened incidence of congenital malformations (69).

To mitigate these risks, it is recommended to discontinue mycophenolate at least 6 weeks before conception (70, 71). Alternatively, azathioprine or tacrolimus can be substituted for mycophenolate before and during pregnancy. Another viable option involves using glucocorticoids at the minimal effective dose for controlling disease activity. Ideally, this transition in medication should be implemented four to six months before the intended conception, ensuring a proactive approach to managing potential adverse outcomes.

### Methotrexate

Administering methotrexate before and during pregnancy carries the potential for lasting consequences on fetal growth (72). To avert adverse effects, it is advised to cease methotrexate use within the window of one to three months preceding conception.

### Leflunomide

It is cautioned to be avoided during pregnancy based on the results of preclinical reproductive studies, although its definitive teratogenicity in humans remains uncertain. For those desiring conception while on this medication, a cholestyramine washout is recommended until blood levels reach undetectable concentrations (73, 74).

### Voclosporin

The utilization of voclosporin in pregnant women is discouraged due to its formulation containing alcohol. The presence of alcohol during pregnancy is correlated with potential adverse effects on fetal development, including abnormalities in the central nervous system and potential compromise of intellectual development (75).

### Anifrolumab

Current data is limited, making it inadequate for a comprehensive assessment of potential drug-related risks, including significant congenital abnormalities, spontaneous pregnancy loss, or unfavorable consequences for both maternal and fetal health (75).

### SGLT2 inhibitors

The role of SGLT2 inhibitors in managing LN presents a nuanced picture. These medications have shown renoprotective effects in lupus models, suggesting potential as non-immunosuppressive agents to enhance renal function in autoimmune kidney diseases like LN (76).

A phase I/II trial indicated that dapagliflozin, an SGLT2 inhibitor, has an acceptable safety profile when used as an add-on therapy in adult SLE patients. However, it did not effectively reduce disease activity or proteinuria in LN patients (77). Their use in pregnancy is cautioned against due to associated risks such as dilation of renal pelvis and tubules, congenital anomalies, and a higher incidence of miscarriages (78). The recommendation is to avoid these drugs during pregnancy and lactation, especially considering the availability of safer alternatives.

## Management during pregnancy

The care of pregnant individuals with LN necessitates a coordinated effort among a rheumatologist, nephrologist, and an obstetrician well-versed in managing high-risk pregnancies. A comprehensive strategy for overseeing pregnant women with LN, along with addressing active LN flare-ups during pregnancy, is outlined in the subsequent sections.

### Monitoring LN activity

We recommend closely monitoring LN activity during pregnancy, necessitating regular assessments by a nephrologist every trimester. For individuals with active SLE, a higher frequency of evaluations is advised. The structured schedule for monitoring comprises the following key elements:

### Initial evaluation

This initial evaluation, as recommended by prior studies (79, 80), should encompass the following investigations:

- Physical examination and vital signs measurement.
- Complete blood count (CBC).
- Kidney function tests (serum creatinine, urinalysis, spot urine protein/creatinine ratio).
- Liver function tests.
- Anti-Ro/SSA and anti-La/SSB antibodies, if not previously assessed.
- Complement (CH50, or C3 and C4).
- Anti-double-stranded DNA (dsDNA) antibodies.
- Antiphospholipid antibodies (lupus anticoagulant [LA], IgG and IgM anticardiolipin [aCL] antibodies, and IgG and IgM anti-beta2 glycoprotein [anti-beta2GPI] antibodies).

During pregnancy, specific physiological alterations can coincide with manifestations of active LN, posing challenges in differentiation. For instance, common laboratory indicators seen in a typical pregnancy involve an increased erythrocyte sedimentation rate (ESR) and proteinuria. Notably, there is an increase in protein excretion during normal pregnancy; however, it should not exceed 300 mg/24 hours. To differentiate between an LN flare, preeclampsia, and routine pregnancy-related alterations in later stages, a baseline 24-hour urine collection or urine protein to creatinine ratio proves valuable, especially after stopping ACE-I/ARB.

Consequently, interpreting laboratory results necessitates a comprehensive consideration of the clinical context. Women exhibiting increased serologic activity without accompanying symptoms require vigilant monitoring. It is not recommended to initiate immunosuppressants based solely on serologic findings.

### Laboratory assessments during pregnancy

Throughout pregnancy in individuals with lupus nephritis, routine laboratory assessments are pivotal for monitoring maternal and fetal well-being. A comprehensive evaluation, including a physical examination with blood pressure measurement, is essential. Regular intervals necessitate the performance of the following laboratory tests:

- Complete Blood Count:
- Creatinine Levels
- Urinalysis with Examination of Urinary Sediment
- Spot Urine Protein/Creatinine Ratio or 24-hour Urine Collection

In cases where active disease is evident or there is a history of prior alterations in associated levels during a flare, additional specific laboratory tests become imperative:

- Anti-dsDNA Antibodies
- Complement Levels (CH50, or C3 and C4): Recent research has identified low C4 levels at preconception as an independent risk factor for SLE flare during pregnancy (81). Low C3 levels have also been shown to be a potential risk factor for preterm delivery in SLE patients (82). This highlights the importance of monitoring the absolute levels of complement components C3 and C4 and their variations over time (81, 82). Understanding that C3/C4 levels naturally increase during pregnancy is crucial. Therefore, baseline levels should be established early in pregnancy. Significant changes in these levels, even if they fall within the normal range for non-pregnant individuals, may indicate a potential flare of SLE. It is crucial to emphasize that the clinical presentation must drive the decision to request additional laboratory assessments, such as assessments for liver function and serum uric acid. The frequency of laboratory testing is personalized and contingent upon the level of disease activity. Ideally, stable patients need testing every trimester. Conversely, individuals with active disease need more frequent testing. Importantly, regular monitoring every 4-8 weeks is recommended due to the utility of observing trends in these parameters over time, aiding in detecting and diagnosing both SLE flare-ups and pre-eclampsia (83).

## Maternal-fetal monitoring

Maternal-fetal monitoring strategies during pregnancy lack a universally optimal schedule. Increased monitoring frequency is warranted for women with SLE in conjunction with routine prenatal care; the monitoring protocol includes:

- A first-trimester ultrasound estimates the delivery date, with a fetal anatomic survey around 18 weeks of gestation (84).
- Ultrasound examinations in the third trimester are utilized to evaluate fetal growth and identify placental insufficiency, with the scheduling, often every four weeks, tailored based on the health of the mother and fetus (84). In cases where there is a suspicion of growth restriction or placental insufficiency, more frequent assessments, including doppler velocimetry, are advised. Furthermore, for late-onset intrauterine growth restriction (IUGR) identified after 34 weeks, signs such as a reduced abdominal circumference, a slowdown in growth velocity, or a low cerebroplacental ratio on doppler studies may signal a higher risk of adverse perinatal outcomes.
- Fetal monitoring, which includes nonstress tests and/or biophysical profiles, is typically recommended for most women with lupus during the last four to six weeks of pregnancy. Surveillance strategies are customized according to continuous fetal and maternal health assessments.
- It is highly recommended that patients who tested positive for anti-Ro/SSA and/or anti-La/SSB antibodies undergo vigilant screening for congenital heart block (85). Women with anti-Ro antibodies linked to neonatal lupus and risk of congenital heart block (ranging from 0.7% to 2%), especially when antibody levels are high, require additional fetal surveillance. The EULAR guidelines recommend fetal echocardiography for suspected cases of fetal dysrhythmia or myocarditis, particularly in those who are positive for Ro/SSA or La/SSB autoantibodies (85).

## Management of LN flares during pregnancy

The diagnosis of lupus nephritis flare during pregnancy necessitates the initiation of therapy (86). A severe lupus nephritis flare, particularly in a young woman early in her pregnancy, should catalyze deliberations regarding therapeutic abortion. Although medical termination of pregnancy might be the safest option to manage lupus optimally, many women, particularly later in gestation or when it may represent their sole opportunity for pregnancy, opt against abortion. Treating LN flare as soon as possible becomes the sensible pathway (84, 87).

## Recommended therapeutic approach

A first-line strategy typically involves steroids. Lupus nephritis usually warrants the addition of immunosuppressive treatment to steroids. A conventional approach is starting azathioprine up to 2mg/kg/day. The utility of calcineurin agent tacrolimus is beneficial due to its steroid-sparing capacity, rapid reduction of proteinuria due to off-target effects on podocyte stabilization, and favorable tolerability. Treatments involving tacrolimus have reported favorable pregnancy outcomes (84, 88).

Furthermore, women with nephrotic range proteinuria should be prescribed prophylactic low-molecular-weight heparin to mitigate venous thromboembolism risk. Tight monitoring in the first six months after giving birth is crucial due to the elevated risk of an LN flare-up (88).

During the later stages of pregnancy, cyclophosphamide could potentially be used to treat severe cases of lupus or LN (89). Nevertheless, the decision to use this treatment involves careful consideration of the risks associated with premature delivery and the subsequent separate management of the health of both mother and baby.

## Preeclampsia

Enhanced monitoring is essential for these patients, especially when symptoms such as hypertension, proteinuria, or end-organ dysfunction emerge after the 20th week of pregnancy, signaling the possible onset of preeclampsia. The occurrence of severe, early-onset fetal growth restriction further signifies an increased risk of developing preeclampsia (90). Although preeclampsia that arises later in pregnancy can typically be managed with a watchful waiting approach, early detection during pre- and peri-viable stages is critical to facilitate prompt delivery and prevent complications in the mother.

The likelihood of developing preeclampsia in individuals with SLE varies from 16 to 30 percent. Low-dose aspirin therapy between the 12th and 16th weeks of pregnancy can decrease the absolute risk of preeclampsia by about 10 percent for those at higher risk, including all patients with SLE (33).

## Preeclampsia versus lupus nephritis

Pre-eclampsia is a severe multisystem disorder of pregnancy characterized by high blood pressure after 20 weeks of gestation, accompanied by signs like proteinuria and maternal or fetal complications (91). Although numerous risk factors for pre-eclampsia have been identified, their ability to predict the condition is generally limited, even when combined. Lupus, especially in its active form with lupus nephritis, significantly increases the risk of pre-eclampsia (92, 93).

Flares of lupus nephritis, while potentially being the initial presentation of lupus, remain relatively rare, particularly in those devoid of prior or inactive nephritis at pregnancy onset. Distinguishing preeclampsia from lupus nephritis or a lupus flare poses a considerable challenge (**Table 3**). During pregnancy, flares of lupus nephritis may resemble preeclampsia, showcasing heightened proteinuria, hypertension, thrombocytopenia, and declining renal function (94). Simultaneous occurrences of active lupus nephritis and preeclampsia are also plausible. If a woman shows signs of increasing proteinuria, hypertension, and deteriorating kidney function early in pregnancy, especially if she has a history of lupus nephritis, this strongly indicates the likelihood of a lupus nephritis flare-up (84, 94).

**Table 3:** Key clinical indicators differentiating preeclampsia from lupus nephritis in pregnancy (94–96).

Clinical and Laboratory Features	Active Lupus Nephritis	Preeclampsia
Symptoms Onset	Anytime during pregnancy	After 20 weeks of pregnancy
Hypertension	+/-	+
Extrarenal SLE signs and symptoms	+	–
Serum uric acid levels	<4.9 mg/dL (low or normal)	>4.9 mg/dL (high)
Complement levels	↓/normal	Normal
Anti ds-DNA levels	↑	Normal
Active urinary sediments	+	–
Urinary calcium excretion	>195 mg/day	<195 mg/day
sFlt-1/PlGF ratio	Normal	↑
Proteinuria	≥300 mg/day	≥300 mg/dl
24-hour urine calcium	≥195 mg/day	<195 mg/day
Complement levels	≥25% drop	Normal

The task of differentiating lupus nephritis from preeclampsia, especially from the late second trimester, becomes markedly complex, as both can manifest with proteinuria, hypertension, generalized symptoms, thrombocytopenia, and kidney impairment. Diagnostic confirmation often leans heavily on historical data and is augmented by suitable investigations. Complement levels should typically be normal or elevated, given the pregnancy’s state of acute phase response. A decrement, even within the normal range, alongside elevating dsDNA antibody levels, should raise alarms regarding lupus activity. A higher soluble fms-like tyrosine kinase-1 (sFLT-1) to placental growth factor (PIGF) ratio in the third trimester was particularly noted in women with preeclampsia compared to those with chronic kidney disease (97). This indicates the possible clinical benefit of assessing the sFlt-1/PIGF ratio in cases of new-onset lupus nephritis and other forms of glomerulonephritis that are associated with hypertension. However, it is essential to note that this diagnostic modality is not yet widely available for routine clinical use.

If there is a notable and unexpected increase in proteinuria that could potentially impact immunosuppressive management, and the woman is in the early or middle stages of the second trimester, a kidney biopsy might be performed, bearing in mind that pregnancy does not increase biopsy risks.

However, consideration must be given to the potential post-biopsy bleeding and the permissible duration for delaying anticoagulation in a pregnant woman with substantial proteinuria and possibly anti-phospholipid antibodies, thus increasing the risk for venous thromboembolism. If thromboembolism risks exceed the diagnostic benefits, biopsy should be forsaken. A biopsy is unnecessary if serology aligns with an LN flare and previous nephritis is documented.

## Delivery and postpartum care

For patients with lupus nephritis, it is crucial to provide advice regarding the likelihood of delivery before 37 weeks and the increased risk of operative delivery if it is very early. The delivery timing should be meticulously determined based on a thorough maternal and fetal well-being evaluation. It is imperative to note that a nephritis flare is not an indication to proceed with delivery.

Postpartum monitoring is imperative, recognizing that many disease flares and thromboembolic events manifest within the initial six months post-delivery (98). Breastfeeding is encouraged; however, a personalized discussion regarding the safety of medications during lactation is crucial. Individual risk assessments are necessary, particularly for premature or ill infants who may face heightened vulnerability to certain medications. Hydroxychloroquine (HCQ), cyclosporine, tacrolimus, prednisone, and azathioprine are considered safe for use during breastfeeding. On the other hand, mycophenolate mofetil, leflunomide methotrexate, and cyclophosphamide are deemed unsafe for breastfeeding mothers. Biologics, except for NFα inhibitors, seem to be safe and effective without a significant maternal or fetal risk and are not recommended during pregnancy and breastfeeding due to insufficient data (99, 100).

## Management of pregnancy after kidney transplant

Pregnancy in women with LN who have undergone a kidney transplant is a feasible option despite presenting specific challenges that necessitate careful preparation. These women can achieve pregnancies that do not jeopardize their transplant’s health, though they do face a higher likelihood of experiencing preeclampsia and miscarriages (101). However, with diligent care and oversight, the health outcomes for both the mother and child can match those of transplant recipients without LN (102). It’s essential to begin preparing for pregnancy between one and two years after the transplant to ensure the kidney is functioning stably without major issues like significant proteinuria, recurrence of LN, uncontrolled hypertension, episodes of rejection, or other graft complications (103). A crucial part of this preparation involves adjusting immunosuppressive treatment, notably discontinuing mycophenolate mofetil before conception to mitigate its risks and potentially switching to azathioprine, a less risky antimetabolite, when necessary (103).

## Risk of cardiovascular complication

Although the risk of cardiovascular complications in patients with lupus nephritis during pregnancy has been less studied, recent research indicates that pregnant patients with SLE face increased risks of acute cardiovascular complications, including peripartum cardiomyopathy, heart failure, arrhythmias, stroke, and venous thromboembolism, during delivery hospitalization (104).

Additionally, these patients are at a higher risk of developing hypertension later and are nearly four times more likely to encounter subclinical cardiovascular diseases in the future, particularly those who have had adverse pregnancy outcomes like pre-eclampsia (105).

Given these risks, it is crucial for women with SLE and lupus nephritis to receive thorough counseling about the potential for acute cardiovascular complications during their delivery hospital stay. This counseling should address not only common comorbidities such as hypertension, diabetes, obesity, and dyslipidemia but also the systemic inflammatory state typical of SLE. Implementing this comprehensive approach is essential to effectively mitigate both immediate and long-term cardiovascular risks for pregnant patients with lupus nephritis.

## Renal replacement therapy

In the management of pregnant women with lupus nephritis and significant renal impairment undergoing renal replacement therapy, several critical interventions are essential to ensure both maternal and fetal well-being. To improve clearance and reduce volume fluctuations, it is recommended that hemodialysis sessions be increased in frequency and intensity, with five to seven sessions per week and a minimum of 24 hours per week (106). This approach has been shown to improve outcomes significantly, reducing pre-dialysis urea levels to below 20 mmol/L, thus mitigating the risk of uremic complications that could affect both mother and child (106, 107).

Ensuring adequate erythropoiesis and managing anemia are equally crucial; increased doses of erythropoiesis-stimulating agents may be required to maintain hemoglobin levels within the desired range, thereby supporting proper fetal growth and reducing risks associated with anemia, such as low birth weight and premature birth (108).

Nutritional management tailored to the needs of renal impairment also plays a pivotal role. High-protein diets are necessary to meet the increased metabolic demands during pregnancy, and adjustments in vitamin and mineral intake, such as calcium and phosphate, are vital (106, 109). Careful selection of phosphate binders like calcium carbonate, which is deemed safe during pregnancy, helps manage serum phosphate levels without exposing the fetus to potential harm (106).

Additionally, daily monitoring and adjustments of fluid balance, electrolytes, and dialysis parameters are necessary to address the physiological changes during pregnancy (106, 107). These measures help prevent complications such as hypercalcemia or metabolic alkalosis, which could otherwise lead to significant maternal and fetal health issues. Thus, a multidisciplinary approach involving nephrologists, obstetricians, and dieticians is imperative to optimize outcomes for both the mother and the developing fetus.

## Conclusions

Pregnancy-induced changes hold the potential to trigger LN flares, impacting maternal health. To optimize pregnancy outcomes for patients with SLE and lupus nephritis, it is crucial to plan conception during periods of disease stability and to ensure the use of medications that are safe during pregnancy. This scenario poses a significant challenge for nephrologists and rheumatologists due to the heightened risk of adverse perinatal outcomes and complications related to pregnancy-induced hypertension. Future efforts in the management of lupus nephritis during pregnancy should emphasize the development of personalized management strategies, underscoring the pressing need for more clinical trials to better understand the safety and efficacy of current SLE treatments during this critical period.

## Author contributions

ZG: Conceptualization, Data curation, Validation, Writing – original draft, Writing – review & editing. TS: Supervision, Validation, Writing – original draft, Writing – review & editing. KJ: Validation, Writing – original draft, Writing – review & editing. SS: Validation, Writing – original draft, Writing – review & editing. EL: Validation, Writing – original draft, Writing – review & editing. AA: Conceptualization, Supervision, Writing – original draft, Writing – review & editing. SN: Conceptualization, Supervision, Validation, Writing – original draft, Writing – review & editing.
